# Well-Aerated Lung and Mean Lung Density Quantified by CT at Discharge to Predict Pulmonary Diffusion Function 5 Months after COVID-19

**DOI:** 10.3390/diagnostics12122921

**Published:** 2022-11-23

**Authors:** Leqing Chen, Feihong Wu, Jia Huang, Jinrong Yang, Wenliang Fan, Zhuang Nie, Hongwei Jiang, Jiazheng Wang, Wenfang Xia, Fan Yang

**Affiliations:** 1 Department of Radiology, Union Hospital, Tongji Medical College, Huazhong University of Science and Technology, Wuhan 430022, China; 2 Hubei Province Key Laboratory of Molecular Imaging, Wuhan 430022, China; 3 Department of Epidemiology and Biostatistics, Ministry of Education Key Laboratory of Environment and Health, School of Public Health, Tongji Medical College, Huazhong University of Science and Technology, Wuhan 430022, China; 4 MSC Clinical & Technical Solutions, Philips Healthcare, Floor 7, Building 2, World Profit Center, Beijing 100600, China; 5 Department of Endocrinology, Union Hospital, Tongji Medical College, Huazhong University of Science and Technology, Wuhan 430022, China

**Keywords:** COVID-19, lung diseases, pulmonary function tests, tomography, X-ray computed, quantitative evaluation

## Abstract

Background: The aim of this study was to explore the predictive values of quantitative CT indices of the total lung and lung lobe tissue at discharge for the pulmonary diffusion function of coronavirus disease 2019 (COVID-19) patients at 5 months after symptom onset. Methods: A total of 90 patients with moderate and severe COVID-19 underwent CT scans at discharge, and pulmonary function tests (PFTs) were performed 5 months after symptom onset. The differences in quantitative CT and PFT results between Group 1 (patients with abnormal diffusion function) and Group 2 (patients with normal diffusion function) were compared by the chi-square test, Fisher’s exact test or Mann–Whitney *U* test. Univariate analysis, stepwise linear regression and logistic regression were used to determine the predictors of diffusion function in convalescent patients. Results: A total of 37.80% (34/90) of patients presented diffusion dysfunction at 5 months after symptom onset. The mean lung density (MLD) of the total lung tissue in Group 1 was higher than that in Group 2, and the percentage of the well-aerated lung (WAL) tissue volume (WAL%) of Group 1 was lower than that of Group 2 (all *p* < 0.05). Multiple stepwise linear regression identified only WAL and WAL% of the left upper lobe (LUL) as parameters that positively correlated with the percent of the predicted value of diffusion capacity of the lungs for carbon monoxide (WAL: *p* = 0.002; WAL%: *p* = 0.004), and multiple stepwise logistic regression identified MLD and MLD_LUL_ as independent predictors of diffusion dysfunction (MLD: OR (95%CI): 1.011 (1.001, 1.02), *p* = 0.035; MLD_LUL_: OR (95%CI): 1.016 (1.004, 1.027), *p* = 0.008). Conclusion: At five months after symptom onset, more than one-third of moderate and severe COVID-19 patients presented with diffusion dysfunction. The well-aerated lung and mean lung density quantified by CT at discharge could be predictors of diffusion function in convalesce.

## 1. Introduction

Since the outbreak of the coronavirus disease 2019 (COVID-19) pandemic in December 2019, caused by severe acute respiratory syndrome coronavirus 2 (SARS-CoV-2) infection, as of November 2022, the World Health Organization (WHO) reported that the number of confirmed patients has exceeded 600 million, with some patients still in convalescence or under treatment. Past experience with severe acute respiratory syndrome (SARS) has shown that residual lung lesions and reduced motor function in convalescent patients will gradually improve over time. However, there are still some sequelae that will last for months or even years, such as pulmonary fibrosis, impaired pulmonary function, etc. In the previous follow-up studies on SARS patients, their pulmonary functional ability capacity and health status were significantly inferior to their normal levels at 6 months after symptom onset [1,2], which indicated the necessity of follow-up investigations of pulmonary functions in COVID-19 patients as a part of public health management.

To date, several studies have reported pulmonary dysfunctions, mainly manifested as diffusion dysfunction, in convalescent COVID-19 patients [3,4,5,6,7]. Diffusion function is the ability of gas to diffuse from the alveoli into the pulmonary capillaries and to bind to hemoglobin [8], which may be affected by the severity of COVID-19 pneumonia during hospitalization [4,5,6,7,8,9]. However, pulmonary function of convalescent patients has remained understudied, partially due to the relatively limited availability of clinical pulmonary function test (PFT) equipment and partially due to the potential risk of cross-infection [10]. A potential alternative to PFT for lung function evaluation is CT, which is more widely accessible during the COVID-19 outbreak and has been proven to provide semiquantitative assessments of pneumonia lesions that were correlated with the severity of COVID-19 [11,12,13]. However, the semiquantitative CT score, a quick and expedient method for disease evaluation in emergency settings, can be influenced by the scoring criteria used and the radiologists’ experience. In contrast, artificial intelligence (AI)-based quantitative analysis of the lesions has shown advantages in disease monitoring and clinical prognosis prediction [14,15,16]. In addition, while PFTs can measure lung functions only at the total lung level, AI can assess volumes and functions of individual lobes [17].

The World Health Organization’s interim guidance diagnostic criteria classify COVID-19 pneumonia into the following four types: mild, moderate, severe and critical [18]. Mild patients usually have a better prognosis, while critical patients deteriorate rapidly, and may experience dyspnea, hypoxemia, ARDS, etc., and the prognosis is poor. Studies have shown that 26% of hospitalized patients require intensive care unit (ICU) care and treatment, and the mortality rate is about 4.3%, while moderate and severe patients account for the majority of hospitalized patients [19]. Therefore, the disease progression and prognosis of moderate and severe COVID-19 patients have received more attention.

In this ambispective observational study, moderate and severe COVID-19 patients were followed for 5 months after symptom onset, and the quantitative CT indices of lung volume and residual lesions at discharge were utilized to predict diffusion dysfunction in COVID-19 survivors to provide optimized management plans for convalescent COVID-19 patients.

## 2. Materials and Methods

### 2.1. Patients

Patients with COVID-19 who were discharged from our hospital between 20 January 2020 and 17 April 2020 were prospectively enrolled. This study was approved by the institutional review boards of the Medical Ethics Committee of our hospital (NO. 0271-01). All participants signed informed consent. The inclusion criteria were as follows: (1) age ≥ 18 years; (2) moderate and severe cases according to the clinical classification standard and the guidelines developed by the WHO (moderate: with clinical signs of pneumonia (fever, cough, dyspnea and tachypnea) but no signs of severe pneumonia, including SpO2 ≥ 90% in room air. Severe: with clinical signs of pneumonia, plus one of the following: respiratory rate > 30 breaths/min; severe respiratory distress; or SpO2 < 90% in room air [18]); and (3) CT scan performed at discharge. The exclusion criteria were as follows: (1) history of tumors or chronic pulmonary diseases; (2) history of lung surgery or thoracic deformity; (3) the quality of CT images did not meet the quantitative analysis requirements; and (4) no PFT was performed at 5 months after symptom onset. The discharge standards were based on the Diagnosis and Treatment Protocol for COVID-19 in China (Trial Fifth Edition) [20].

The baseline data were collected from the electronic medical record system, including age, sex, height, weight, comorbidities, time of symptom onset, and length of hospital stay.

### 2.2. Chest CT Scan Protocol and AI-Based Quantitative Data Acquisition

All patients underwent CT scans using the IQon Spectral CT (Philips Healthcare, Best, The Netherlands) or SOMATOM Definition AS+ (Siemens Healthineers. Forchheim, Germany) in the supine position following deep inspiration (median duration from symptom onset to chest CT: 32 days [14, 40]). The scan covered the thoracic inlet to the diaphragm and the instrument settings were as follows: (1) a fixed tube voltage of 120 kV; (2) adaptive tube current; (3) a pitch of 0.984 with a detector (64 × 0.625 mm); (4) slice thicknesses of 1.25 mm or 2.00 mm and intervals of 1.25 mm or 2.00 mm. All images were reconstructed with lung and soft tissue kernels and stored in the local picture archiving and communication system (PACS).

**Figure 1:**
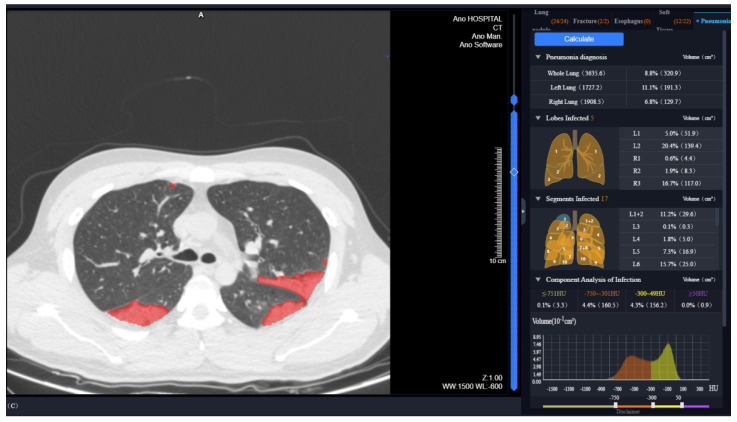
Quantitative analysis of CT images at discharge in uAI intelligent assistant system.

Two radiologists (with 5 and 27 years of experience) recorded the lesion locations present on the CT images at discharge in a consistent manner. Quantitative analysis of the lung and lobar volumes and the lesions was performed on the uAI Intelligent Assistant Analysis System (United Imaging Intelligence), as previously reported [17,21]. Each lobe was automatically segmented by identifying the bilateral lung contours and the interlobular fissures (left oblique fissure, right horizontal and oblique fissure), and the pneumonia lesions were distinguished automatically. Manual correction was performed by one radiologist in case of inaccurate delineation, and rechecked by another radiologist. Well-aerated lung tissue (−950 HU to −750 HU), ground-glass opacity (GGO) lesions (−750 HU to −300 HU), and consolidation lesions (−300 HU to 50 HU) were distinguished based on the CT values [22,23,24]. In total, the following parameters related to the volume and residual lesions in the entire lung and five lobes were measured (Figure 1): (1) mean lung density of total lung tissue (or lobe tissue) (MLD, HU); (2) total lung (or lobar) volume (LV, cm^3^); (3) well-aerated lung tissue volume (WAL, cm^3^); (4) mean lung density of lesion (MLD_Le_, HU); (5) total lung (or lobar) lesion volume (LeV, cm^3^); (6) GGO volume (GV, cm^3^); (7) consolidation volume (CV, cm^3^); (8) percentage of lobar volume to total lung volume (LV%); (9) percentage of LeV to total lung (or lobar) volume (LeV%); (10) percentage of the WAL to total lung (or lobar) volume (WAL%); (11) percentage of GV of the lesion (GV%); and (12) percentage of CV of the lesion (CV%).

### 2.3. Pulmonary Function Tests (PFTs)

All PFTs were performed by 2 experienced technicians using Master Lab equipment (CareFusion, Hoechberg, Germany), following the American Thoracic Society Committee criteria (median duration from symptom onset to PFTs: 146 [140, 164] days) [25]. The following parameters were recorded: forced expiratory volume in one second (FEV1), forced vital capacity (FVC), maximal instantaneous forced expiratory flow, where 75% and 50% of the FVC remain to be expired (MEF75% and MEF50%), maximum mid-expiratory flow (MMEF), total lung capacity (TLC), diffusion capacity of the lungs for carbon monoxide (DL_CO_) and alveolar ventilation (VA). In addition, the FEV1/FVC ratio and DL_CO_/VA ratio were calculated, and all the results were expressed as a percentage of the predicted value. The diffusion function was considered abnormal if DL_CO_ < 80% of the predicted value.

### 2.4. Statistical Analysis

Statistical analysis was performed using SAS software (SAS, version 9.4, SAS Institute). Categorical variables are presented as numbers (%), and continuous variables are presented as medians (Q1 and Q3). All the COVID-19 patients in convalescence were divided into Group 1 (patients with abnormal diffusion function) and Group 2 (patients with normal diffusion function), according to whether their pulmonary diffusion function was impaired at 5 months after symptom onset. Demographic characteristics, COVID-19 clinical classification, duration of hospital stay, and the quantitative CT indices of lung volume and residual lesions were compared between Group 1 and Group 2. Categorical variables were assessed by the chi-square test or Fisher’s exact test, and continuous variables were assessed by the Mann–Whitney *U* test. Univariate analysis and stepwise linear regression were used to determine the predictors of DL_CO_%, and univariate analysis and stepwise logistic regression were used to determine risk factors for diffusion dysfunction in convalescent COVID-19 patients. Indicators with *p <* 0.05 were included in the equation, while those with *p* > 0.1 were excluded. A receiver operating characteristic (ROC) curve was analyzed for the final model of diffusion dysfunction prediction, and maximization of the Youden index was selected as the optimal criterion for selecting the cutoff point to assess the performance of the model. Calibration was assessed by comparing the agreement between the observed and predicted numbers of diffusion dysfunctions in the groups stratified according to deciles of predicted risk using the Hosmer–Lemeshow (HL) test and calibration plots. Statistical significance was set at *p* < 0.05 (two-tailed).

## 3. Results

### 3.1. Characteristics of COVID-19 Patients

**Figure 2:**
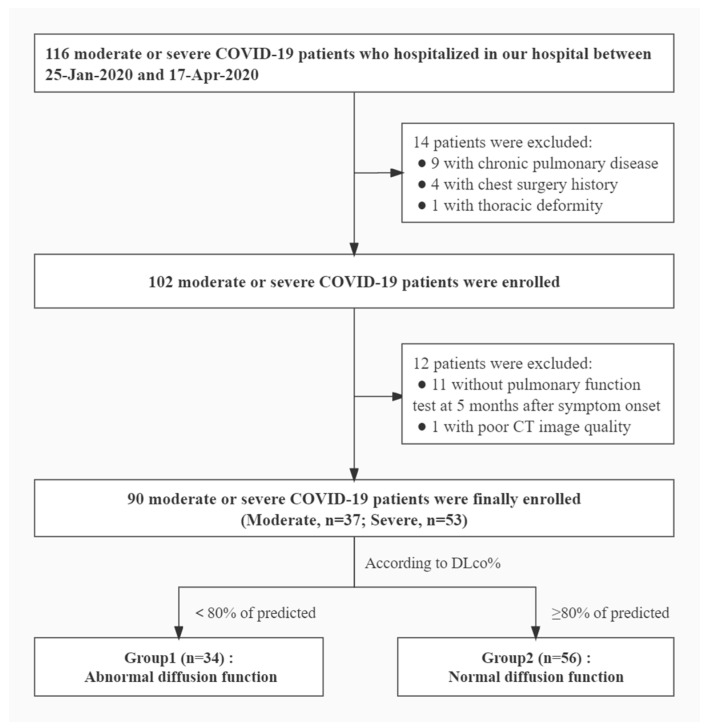
Flowchart of patient inclusion.

A total of 116 patients with moderate and severe COVID-19 were initially included in this study, of whom 26 patients were excluded for the following reasons: 11 patients did not undergo PFTs at 5 months after symptom onset, 9 patients had a history of chronic pulmonary diseases (emphysema and tuberculosis), 4 patients had a history of chest surgery, 1 patient had a thoracic deformity, and the quality of 1 patient’s CT image was too poor due to motion artifacts. Finally, 90 patients that underwent PFTs at 5 months from symptom onset were included (median duration from symptom onset to discharge: 32 [14, 40] days). The flow chart of patient enrollment is shown in Figure 2.

In this study, 90 patients (35 male, 55 female; median age: 57.00 (49.75, 64.25) years) that underwent PFTs at 5 months from symptom onset were included, comprising 34 patients with abnormal diffusion function in Group 1 (12 male, 22 female; median age: 59.00 (49.75, 67.00) years) and 56 patients with normal diffusion function in Group 2 (23 male, 33 female; median age: 56.00 (47.75, 63.00) years). There were no differences in baseline demographic characteristics, comorbidities, clinical types, or the duration of hospital stay between the two groups (Table 1, all *p* > 0.05).

### 3.2. CT Findings and Quantitative Results of COVID-19 Survivors at Discharge

The total lung tissue and lobar measurements from the CT scans at discharge are shown in Table 2 and Table S1, respectively. Only 10 (11.10%) of the 90 patients had pneumonia lesions that had been completely absorbed at discharge (1 in Group 1; 9 in Group 2). There were no differences in the distribution of residual lesions among lobes between the two groups (all *p* > 0.05, Table 2). The MLD of the total lung tissue of Group 1 was higher than that of Group 2 (Group 1: −803.60 HU (−823.48 HU, −752.50 HU), Group 2: −815.75 HU (−832.88 HU, −796.33 HU), *p* = 0.023), and the WAL% of Group 1 was lower than that of Group 2 (Group 1: 79.38% (68.37%, 83.03%), Group 2: 83.18% (76.49%, 85.66%), *p* = 0.019), which was mainly due to the differences in the bilateral upper lobe and the right middle lobe (RML). In the comparison of the lesion composition, except for the percentage of consolidation of the left upper lobe (CV_LUL_%), there were no differences in the proportion of different lesion components between the two groups (all *p* > 0.05, Table 2; Table S1).

### 3.3. Pulmonary Function of COVID-19 Survivors at 5 Months after Symptom Onset

A total of 53 out of 90 (58.90%) patients reported impaired pulmonary function at 5 months after symptom onset, of whom 34 patients had abnormal diffusion function (DL_CO_% of predicted value: 74.55% (65.73%, 75.73%), Table 3). Among the spirometry-based indices, only FEV1% of the predicted value was significantly different between the two groups (Group 1: 89.10% (80.38%, 111.93%), Group 2: 100.60% (92.95%, 109.35%), *p* = 0.011). Among the lung volume indices, TLC% of the predicted value and FVC% of the predicted value were both significantly lower in Group 1 than in Group 2 (both *p* < 0.05), and there were significantly more patients with impaired TLC% of the predicted value in Group 1 than in Group 2 (Group 1: 35.30%; Group 2: 3.60%, *p* < 0.001).

### 3.4. Quantitative CT Factors Associated with Abnormal Diffusion Function at 5 Months after Symptom Onset

The results of the univariate linear analysis that was carried out to determine the predictors of DL_CO_% using the total lung indices and lobar indices showed that the MLD, LeV, LeV%, and the GV of the total lung or bilateral upper lobe tissue were negatively correlated with DL_CO_%, while LV, WAL, and WAL% were positively correlated with DL_CO_% (all *p* < 0.05). However, multiple stepwise linear regression selected only WAL (coefficient: 0.004; 95% CI: 0.001–0.007; *p* = 0.002, Table 4) out of the total lung indices and WALLUL% out of the lobar indices (coefficient: 0.459; 95% CI: 0.152–0.766; *p* = 0.004, Table S2, Figure S1).

**Figure 3:**
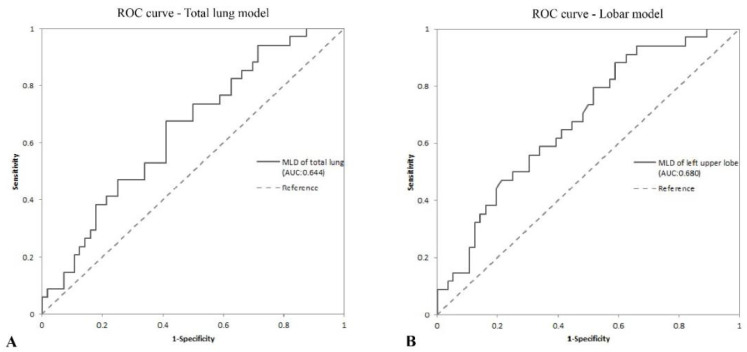
The receiver operating characteristic (ROC) curves of regression models of abnormal diffusion function prediction: (**A**) the ROC curve of total lung model; (**B**) the ROC curve of lobar model.

Multiple stepwise logistic regression (used to predict whether there was diffusion dysfunction in patients) identified only higher MLD (OR: 1.011; 95% CI: 1.001–1.02; *p* = 0.035, Table 4) and MLD_LUL_ (OR: 1.016; 95% CI: 1.004–1.027; *p* = 0.008, Table S3) as risk factors for COVID-19 patients with abnormal diffusion function in convalescence from the total lung and lobar CT indices, respectively. The area under the ROC curve (AUC) estimated for the total lung model was 0.644 (95% CI: 0.528–0.760; Table 5, Figure 3A), with a cutoff value of −810.70 HU of MLD, and the sensitivity and specificity values were 67.6% and 58.9%, respectively. For the lobar model, the AUC was 0.680 (95% CI: 0.568–0.791; Table 5, Figure 3B) with a cutoff value of −837.80 HU of MLD_LUL_, and the sensitivity and specificity values were 88.2% and 41.1%, respectively. The HL test demonstrated a good model fit for the MLD of the total lung tissue (*p* = 0.664) and MLD_LUL_ (*p* = 0.708) values. Calibration plots are reported in Figures S2 and S3.

## 4. Discussion

In this study, we focused on pulmonary diffusion function in convalescent COVID-19 survivors and demonstrated the predictive value of CT imaging at discharge for pulmonary diffusion function at 3 months after discharge. CT imaging at discharge was selected for the prediction study instead of imaging upon admission to reduce selection bias because the former time point is better defined in the disease course. At this time point, all discharge standards have been met (clinical and imaging evaluations, two consecutive negative results from nucleic acid tests, etc.). In contrast, the time point of admission is usually measured against the symptom onset, which tends to be subjective and could be considerably varied in the disease course between patients, particularly considering the rapid development of pulmonary lesions in the early stage of COVID-19 [26,27]. In our 5-month follow-up of the moderate and severe COVID-19 patients, 37.80% (34/90) of the patients presented with abnormal diffusion function, and quantitative CT indices at discharge (particularly MLD of total lung or left upper lung tissue) could be independent predictors of diffusion dysfunction in convalescent patients.

A previous study reported that 21.3% of recovered SARS patients had diffusion dysfunction up to 3 months after discharge [28]. Qin et al. [3] and Huang et al. [5] reported that 54.00% (44/81) and 34.10% (114/334) of COVID-19 patients had diffusion dysfunction at 3 months after discharge and 6 months after symptom onset, respectively. Our study showed that 37.80% (34/90) of patients presented with diffusion dysfunction at 5 months from symptom onset, which was slightly higher than Huang’s estimation, but lower than Qin’s report, and the differences may be caused by the difference in follow-up length.

Our study also showed that the clinical classification of COVID-19 severity and the length of hospital stay were not related to the presence of abnormal diffusion function in convalescent patients, which was different from the findings of Mo et al. [4] and Huang et al. [9]. A potential cause of this discrepancy may be because only moderate and severe patients were included in this study, and patients with mild and critical cases of COVID-19 were not enrolled. There were more patients with reduced total lung capacity in Group 1 than in Group 2 (Table 2), but there was no difference in total lung volume measured by quantitative CT between the two groups at discharge (*p* = 0.276). This may suggest that the total lung volume of convalescent patients with abnormal diffusion function recovered more slowly than that of patients with normal diffusion function.

Previous studies have reported that very low CT threshold values of lung tissue below −950 HU can be pathologically confirmed as emphysema [29], and that areas above −750 HU or −700 HU were considered lesions (e.g., GGOs, consolidation) [23,24]. The lung tissue between −950 HU and −750 HU is considered to be well-aerated lung tissue [30,31,32], which has remained understudied thus far for COVID-19 patients. Colombi et al. [31] analyzed the volume between −950 HU and −700 HU of COVID-19 patients at admission and concluded that the reduced volume of well-aerated lungs predicted adverse outcomes (intensive care unit admission or death). In our study, only the WAL was identified as a predictor of DL_CO_% in convalescent COVID-19 patients by multiple linear regression analysis, with higher WAL indicating higher DL_CO_%. Matsuoka et al. [30] reported that a high attenuation area (CT threshold value: −700 HU~0 HU) was more closely related to DL_CO_%. Our results excluded the lesions (CT threshold value: > −750 HU) by using a more detailed classification system based on CT threshold values, suggesting that DL_CO_%, as an index for evaluating diffusion function, may be more relevant to non-lesion tissues. Therefore, for patients with COVID-19 or other types of pneumonia, CT quantification of non-lesion tissues may be important for the estimation of DL_CO_%, which is different from the routine analysis of the pulmonary lesion or the overall pulmonary state.

An autopsy study reported that the pathological damages to the lungs caused by SARS-CoV-2 were examples of severe endothelial injury associated with the disruption of intercellular junctions, cell swelling, and the loss of contact with the basal membrane [33], which implied that the impaired diffusion function might be mainly due to the membrane damage that affected the gas exchange barrier. On chest CT, the MLD can reflect the gas content, blood distribution, and lung parenchyma [34,35]. In this study, the only risk factor for diffusion dysfunction selected by multiple logistic regression was the MLD, and the risk of diffusion dysfunction in convalesce patients increased when MLD_LUL_ was higher than −837.80 In addition, compared with the total lung and lobar models, the AUC of the lobar model was higher than the AUC of the total lung model (lobar model: 0.680; total lung tissue: 0.644), indicating that the quantitative analysis of CT data from the lobar perspective could achieve a higher prediction accuracy.

Although the initial lesions were mostly in the lower lobes [36,37], previous studies have reported a positive correlation between the consolidation volume in the upper lobes and adverse outcomes [38]. In our study, for the diffusion function of patients in convalescence, univariate analysis identified several indicators that are based on measurements from the bilateral upper lobes, while multivariate stepwise logistic regression analysis identified only the CT indices of the left upper lobes, indicating that the left upper lobe was unique in predicting the diffusion dysfunction in convalescence, which is worth further exploration and analysis.

There are several limitations in our study. First, the sample size of this study was limited, and only moderate and severe COVID-19 patients were enrolled for the follow-up. Patients with these two types of disease are more common than those with critical disease and are more likely to generate pressures on public health management than patients with mild disease. Second, PFTs were not performed at discharge; therefore, the chronological changes in diffusion function could not be monitored, and the quantitative CT indices at discharge were not directly compared to PFT indices. Thirdly, our laboratory results were not included in the regression analysis. In addition, a longer follow-up time is required to study whether the quantitative CT indices at discharge can predict the diffusion function of patients in the long term.

## 5. Conclusions

In conclusion, this study reported the predictive values of quantitative CT indices at discharge for the diffusion dysfunction of moderate and severe COVID-19 survivors in convalescence 5 months after symptom onset, among which the mean lung density and percentage of the well-aerated lung tissue volume of the left upper lobe were closely related to diffusion dysfunction. These findings may help with the follow-up management of COVID-19 patients in convalescence.

## Figures and Tables

**Table 1:** The demographics and clinical characteristics in COVID-19 patients.

Characteristics	All Patients (*n* = 90)	Group 1, Abnormal Diffusion Function (*n* = 34)	Group 2, Normal Diffusion Function (*n* = 56)	*p* Value
Age, years	57.00 (49.75, 64.25)	59.00 (49.75, 67.00)	56.00 (47.75, 63.00)	0.275
≤50	24/90 (26.7%)	9/34 (26.5%)	15/56 (26.8%)	0.974
>50	66/90 (73.3%)	25/34 (73.5%)	41/56 (73.2%)	
Sex				
Male	35/90 (38.9%)	12/34 (35.3%)	23/56 (41.1%)	0.586
Female	55/90 (61.1%)	22/34 (64.7%)	33/56 (58.9%)	
Height, cm	163.00 (158.75, 170.00)	164.50 (160.00, 170.00)	161.50 (158.00, 169.75)	0.499
Weight, kg	64.00 (58.00, 72.25)	63.00 (58.00, 71.50)	64.50 (58.00, 74.25)	0.881
BMI, kg/m²	23.96 (22.18, 25.41)	23.34 (21.92, 25.45)	24.02 (22.72, 25.44)	0.495
Comorbidities				
Hypertension	10/90 (11.1%)	6/34 (17.6%)	4/56 (7.1%)	0.124
Diabetes	4/90 (4.4%)	3/34 (8.8%)	1/56 (1.8%)	0.116
Coronary heart disease	2/90 (2.2%)	1/34 (2.9%)	1/56 (1.8%)	0.718
Liver history	5/90 (5.6%)	3/34 (8.8%)	2/56 (3.6%)	0.292
Kidney history	1/90 (1.1%)	1/34 (2.9%)	0/56 (0%)	0.197
Clinical classification				
Moderate	37/90 (41.1%)	12/34 (35.3%)	25/56 (44.6%)	0.382
Severe	53/90 (58.9%)	22/34 (64.7%)	31/56 (55.4%)	
Hospital stay duration, days	32 (14, 40)	33.5 (13.25, 44.75)	31.5 (14.25, 38.75)	0.386

Note: Data are presented as medians (Q1 and Q3) or n/N (%). *p* values that compare Group 1 and Group 2 were obtained from χ² test, Fisher’s exact test or Mann–Whitney *U* test. Abbreviations: BMI: body mass index.

**Table 2:** The total lung tissue quantitative CT indexes of COVID-19 patients at discharge.

Characteristics	All Patients (*n* = 90)	Group1, Abnormal Diffusion Function (*n* = 34)	Group2, Normal Diffusion Function (*n* = 56)	*p* Value
MLD, HU	−810.35 (−829.05, −778.78)	−803.6 (−823.48, −752.5)	−815.75 (−832.88, −796.33)	0.023 *
LV, cm^3^	3978.3 (3524.8, 4576.38)	3978.45 (3429.48, 4334.05)	3971.35 (3526.6, 4805.35)	0.276
Well-aerated lung tissue				
WAL, cm^3^	3203.9 (2641.35, 3830.05)	3130.75 (2298.55, 3547.93)	3310.7 (2799.98, 4022.8)	0.101
WAL%	82.15 (72.59, 85.32)	79.38 (68.37, 83.03)	83.18 (76.49, 85.66)	0.019 *
MLD_Le_, HU	−679.95 (−739.3, −592.83)	−687 (−738, −642.78)	−674.85 (−740.1, −527.25)	0.221
LeV, cm^3^	150.35 (18.9, 389.9)	244.1 (36.38, 608.25)	118.3 (13.23, 331.4)	0.092
LeV%	3.35 (0.5, 10.75)	5.4 (0.83, 16.08)	3 (0.35, 8.73)	0.080
GGO				
GV, cm^3^	64.1 (10.45, 163.98)	100.7 (18.03, 345.93)	50.55 (7.38, 143.95)	0.105
GV%	45.74 (34.42, 59.99)	45 (34.57, 61.36)	45.8 (33.37, 59.53)	0.552
Consolidation				
CV, cm^3^	4.2 (0.6, 20.55)	6.05 (1.08, 25.68)	3.05 (0.43, 17.73)	0.191
CV%	3.18 (1.26, 5.99)	3.43 (1.84, 5.87)	2.8 (1.18, 6.15)	0.407
With residual lesions, n/N (%)	80/90 (88.9%)	33/34 (97.1%)	47/56 (83.9%)	0.055
Location of residual lesion ^a^				
Unilateral, n/N (%)	2/80 (2.5%)	0/33 (0%)	2/47 (4.3%)	0.230
Bilateral, n/N (%)	78/80 (97.5%)	33/33 (100%)	45/47 (95.7%)	
Left upper lobe (LUL), n/N (%)	76/80 (95%)	32/33 (97%)	44/47 (93.6%)	0.498
Left lower lobe (LLL), n/N (%)	78/80 (97.5%)	32/33 (97%)	46/47 (97.9%)	0.799
Right upper lobe (RUL), n/N (%)	74/80 (92.5%)	32/33 (97%)	42/47 (89.4%)	0.203
Right middle lobe (RML), n/N (%)	65/80 (81.3%)	29/33 (87.9%)	36/47 (76.6%)	0.203
Right lower lobe (RLL), n/N (%)	78/80 (97.5%)	33/33 (100%)	45/47 (95.7%)	0.230

Note: Data are presented as medians (Q1 and Q3) or n/N (%). *p* values that compare Group 1 and Group 2 were obtained from *χ²* test, Fisher’s exact test or Mann–Whitney *U* test. ^a^: Analysis results obtained from the patients with residual lesions on CT at discharge (total: *n* = 80; Group1: *n* = 33; Group2: *n* = 47); * *p* < 0.05. Abbreviations: MLD: mean lung density; LV: total lung volume; WAL: well-aerated lung tissue volume; WAL%: percentage of the well-aerated lung volume to the total lung volume; MLD_Le_: mean lung density of lesion; LeV: total lung lesion volume; LeV%: percentage of lesion volume to total lung volume; GV: ground-glass opacity volume; GV%: percentage of ground-glass opacity volume of the lesion; CV: consolidation volume; CV%: percentage of consolidation volume of the lesion.

**Table 3:** Pulmonary function tests of convalescent COVID-19 patients 5 months after symptom onset.

Characteristics	All Patients (*n* = 90)	Group 1, Abnormal Diffusion Function (*n* = 34)	Group 2, Normal Diffusion Function (*n* = 56)	*p* Value
Time from PFTs to symptom onset (days)	146 (140, 164)	150 (141, 165.25)	146 (139.25, 153.75)	0.244
Gas diffusion				
DL_CO_ (% of pred. value)	84.6 (75.53, 93.7)	74.55 (65.73, 75.73)	91.45 (85.28, 98.95)	<0.001 **
<80% of pred. value, n/N (%)	34/90 (37.8%)	34/34 (100%)	0/56 (0%)	
DL_CO_/VA (% of pred. value)	95.7 (85.25, 105.15)	84.95 (77.08, 97.75)	100.65 (90.6, 106.58)	<0.001 **
<80% of pred. value, n/N (%)	13/90 (14.4%)	13/34 (38.2%)	0/56 (0%)	<0.001 **
Spirometry				
FEV1/FVC (%)	76.54 (71.68, 80.45)	78.75 (71.03, 81.17)	76.42 (71.94, 79.69)	0.506
<70%, n/N (%)	17/90 (18.9%)	8/34 (23.5%)	9/56 (16.1%)	0.381
FEV1 (% of pred. value)	97.05 (88.15, 110.25)	89.1 (80.38, 111.93)	100.6 (92.95, 109.35)	0.011 *
<80% of pred. value, n/N (%)	7/90 (7.8%)	5/34 (14.7%)	2/56 (3.6%)	0.056
MEF75% (% of pred. value)	100.15 (81.63, 121.45)	102.9 (75.9, 122.1)	100.15 (85.3, 121.2)	0.677
<65% of pred. value, n/N (%)	4/90 (4.4%)	2/34 (5.9%)	2/56 (3.6%)	0.606
MEF50% (% of pred. value)	75.40 (55.03, 97.93)	79.95 (49.38, 100.43)	71.55 (57, 90.38)	0.894
<65% of pred. value, n/N (%)	33/90 (36.7%)	14/34 (41.2%)	19/56 (33.9%)	0.489
MMEF (% of pred. value)	64.4 (47.23, 81.43)	66.5 (41.78, 90.35)	63.45 (50.53, 79.9)	0.884
<65% of pred. value, n/N (%)	46/90 (51.1%)	17/34 (50%)	29/56 (51.8%)	0.869
Lung volume				
TLC (% of pred. value)	93.2 (84.03, 101.2)	86.8 (75.13, 92.95)	96.45 (87.83, 102.73)	<0.001 **
<80% of pred. value, n/N (%)	14/90 (15.6%)	12/34 (35.3%)	2/56 (3.6%)	<0.001 **
FVC (% of pred. value)	107.15 (96.25, 118.93)	101.35 (90.75, 112.1)	110.05 (101.8, 118.98)	0.014 *
<80% of pred. value, n/N (%)	1/90 (1.1%)	1/34 (2.9%)	0/56 (0%)	0.197

Note: Data are presented as medians (Q1 and Q3) or n/N (%). *p* values that compare Group 1 and Group 2 were obtained from *χ*² test, Fisher’s exact test or Mann–Whitney *U* test. * *p* < 0.05, ** *p* < 0.001. Abbreviations: PFTs: pulmonary function tests; FEV1: forced expiratory volume in one second; FVC: forced vital capacity; MEF: maximal expiratory flow; MMEF: maximal mid-expiratory flow; TLC: total lung capacity; DL_CO_: diffusing capacity of the lungs for carbon monoxide; VA: alveolar ventilation.

**Table 4:** Regression analysis of predictors from total lung tissue quantitative CT indexes of pulmonary diffusion function in convalescent COVID-19 patients.

Variables	Univariable Analysis			Multivariable Analysis		
	Coefficient (95% CI)	OR (95% CI)	*p* Value	Coefficient (95% CI)	OR (95% CI)	*p* Value
Linear regression analysis						
MLD, HU	−0.075 (−0.133, −0.018)	/	0.011 *	/	/	/
LV, cm^3^	0.004 (0.001, 0.007)	/	0.008 *	/	/	/
WAL, cm^3^	0.004 (0.001, 0.007)	/	0.002 *	0.004 (0.001, 0.007)	/	0.002 *
WAL%	0.331 (0.075, 0.588)	/	0.012 *	/	/	/
MLD_Le_, HU	0.007 (−0.005, 0.02)	/	0.234	/	/	/
LeV, cm^3^	−0.01 (−0.017, −0.002)	/	0.011 *	/	/	/
LeV%	−0.377 (−0.635, −0.12)	/	0.005 *	/	/	/
GV, cm^3^	−0.016 (−0.029, −0.002)	/	0.025 *	/	/	/
GV%	−0.065 (−0.2, 0.071)	/	0.347	/	/	/
CV, cm^3^	−0.043 (−0.091, 0.005)	/	0.075	/	/	/
CV%	−0.224 (−0.639, 0.19)	/	0.286	/	/	/
Logistic regression analysis						
MLD, HU	0.01 (0.001, 0.02)	1.011 (1.001, 1.02)	0.035 *	0.01 (0.001, 0.02)	1.011 (1.001, 1.02)	0.035 *
LV, cm^3^	0 (−0.001, 0)	1 (0.999, 1)	0.109	/	/	/
WAL, cm^3^	0 (−0.001, 0)	1 (0.999, 1)	0.046 *	/	/	/
WAL%	−0.042 (−0.082, −0.001)	0.959 (0.921, 0.999)	0.045 *	/	/	/
MLD_Le_, HU	−0.002 (−0.005, 0)	0.998 (0.995, 1)	0.077	/	/	/
LeV, cm^3^	0.001 (0, 0.002)	1.001 (1, 1.002)	0.058	/	/	/
LeV%	0.044 (0.003, 0.086)	1.045 (1.003, 1.09)	0.036 *	/	/	/
GV, cm^3^	0.002 (0, 0.004)	1.002 (1, 1.004)	0.111	/	/	/
GV%	0.013 (−0.008, 0.035)	1.013 (0.992, 1.035)	0.217	/	/	/
SCV, cm^3^	0.007 (−0.007, 0.02)	1.007 (0.993, 1.021)	0.320	/	/	/
SCV%	0.008 (−0.053, 0.069)	1.008 (0.948, 1.071)	0.801	/	/	/

* *p* < 0.05. Abbreviations: OR: odds ratio; CI: confidence interval; MLD: mean lung density; LV: total lung volume; WAL: well-aerated lung tissue volume; WAL%: percentage of the well-aerated lung volume to the total lung volume; MLD_Le_: mean lung density of lesion; LeV: total lung lesion volume; LeV%: percentage of lesion volume to total lung volume; GV: ground-glass opacity volume; GV%: percentage of ground-glass opacity volume of the lesion; CV: consolidation volume; SCV%: percentage of consolidation volume of the lesion.

**Table 5:** Sensitivity, specificity, PPV and NPV with cut-off values of multiple logistic regression models.

	AUC (95% CI)	*p* Value	Cut-Off	Sensitivity (%)	Specificity (%)	PPV (%)	NPV (%)
Total lung model							
MLD_TL_, HU	0.644 (0.528, 0.760)	0.007 *	−810.7	67.6	58.9	50	75
Lobar model							
MLD_LUL_, HU	0.680 (0.568, 0.791)	<0.001 **	−837.8	88.2	41.1	47.6	85.2

* *p* < 0.05, ** *p* < 0.001. Abbreviations: AUC: area under ROC curve; CI: confidence interval; PPV: positive predictive value; NPV: negative predictive value; MLD_TL_: mean lung density of total lung tissue; MLD_LUL_: mean lung density of left upper lobes.

## Data Availability

The data that support the findings of this study are available from the corresponding author upon reasonable request.

## Supplementary Materials

The following supporting information can be downloaded at: https://www.mdpi.com/article/10.3390/diagnostics12122921/s1, Figure S1: Residual plots of linear model for assumptions of normality, linearity and homogeneity of variance; Figure S2: Calibration plot of logistic model on mean lung density of total lung tissue; Figure S3: Calibration plot of logistic model on mean lung density of left upper lobes; Table S1: The lobar quantitative CT indices of COVID-19 patients at discharge; Table S2: Linear regression analysis between lobar quantitative CT indices and DLCO% of predicted values; Table S3: Logistic regression analysis of predictors from lobar quantitative CT indices of diffusion dysfunction in convalescent COVID-19 patients.

## References

[1] Hui D.S., Joynt G.M., Wong K.T., Gomersall C.D., Li T.S., Antonio G., Ko F.W., Chan M.C., Chan D.P., Tong M.W. (2005). Impact of severe acute respiratory syndrome (SARS) on pulmonary function, functional capacity and quality of life in a cohort of survivors. Thorax.

[2] Ngai J.C., Ko F.W.S., Ng S., To K.-W., Tong M., Hui D.S. (2010). The long-term impact of severe acute respiratory syndrome on pulmonary function, exercise capacity and health status. Respirology.

[3] Qin W., Chen S., Zhang Y., Dong F., Zhang Z., Hu B., Zhu Z., Li F., Wang X., Wang Y. (2021). Diffusion capacity abnormalities for carbon monoxide in patients with COVID-19 at Three-Month follow-up. Eur. Respir. J..

[4] Mo X., Jian W., Su Z., Chen M., Peng H., Peng P., Lei C., Chen R., Zhong N., Li S. (2020). Abnormal pulmonary function in COVID-19 patients at time of hospital discharge. Eur. Respir. J..

[5] Huang C., Huang L., Wang Y., Li X., Ren L., Gu X., Kang L., Guo L., Liu M., Zhou X. (2021). 6-Month consequences of COVID-19 in patients discharged from hospital: A cohort study. Lancet.

[6] Wu X., Liu X., Zhou Y., Yu H., Li R., Zhan Q., Ni F., Fang S., Lu Y., Ding X. (2021). 3-Month, 6-month, 9-month, and 12-month respiratory outcomes in patients following COVID-19-related hospitalisation: A prospective study. Lancet Respir. Med..

[7] Blanco J.R., Cobos-Ceballos M.J., Navarro F., Sanjoaquin I., Arnaiz D.L.R.F., Bernal E., Buzon-Martin L., Viribay M., Romero L., Espejo-Perez S. (2021). Pulmonary long-term consequences of COVID-19 infections after hospital discharge. Clin. Microbiol. Infect..

[8] Graham B.L., Brusasco V., Burgos F., Cooper B.G., Jensen R., Kendrick A., MacIntyre N.R., Thompson B.R., Wanger J. (2017). 2017 ERS/ATS standards for single-breath carbon monoxide uptake in the lung. Eur. Respir. J..

[9] Huang Y., Tan C., Wu J., Chen M., Wang Z., Luo L., Zhou X., Liu X., Huang X., Yuan S. (2020). Impact of coronavirus disease 2019 on pulmonary function in early convalescence phase. Respir. Res..

[10] Burgos F., Torres A., González J., Puig De La Bellacasa J., Rodriguez-Roisin R., Roca J. (1996). Bacterial colonization as a potential source of nosocomial respiratory infections in two types of spirometer. Eur. Respir. J..

[11] Li L., Yang L., Gui S., Pan F., Ye T., Liang B., Hu Y., Zheng C. (2020). Association of clinical and radiographic findings with the outcomes of 93 patients with COVID-19 in Wuhan, China. Theranostics.

[12] Xiong Y., Sun D., Liu Y., Fan Y., Zhao L., Li X., Zhu W. (2020). Clinical and High-Resolution CT features of the COVID-19 infection. Investig. Radiol..

[13] Francone M., Iafrate F., Masci G.M., Coco S., Cilia F., Manganaro L., Panebianco V., Andreoli C., Colaiacomo M.C., Zingaropoli M.A. (2020). Chest CT score in COVID-19 patients: Correlation with disease severity and short-term prognosis. Eur. Radiol..

[14] Zhang K., Liu X., Shen J., Li Z., Sang Y., Wu X., Zha Y., Liang W., Wang C., Wang K. (2020). Clinically applicable AI system for accurate diagnosis, quantitative measurements and prognosis of COVID-19 pneumonia using computed tomography. Cell.

[15] Murphy K., Smits H., Knoops A.J.G., Korst M.B.J.M., Samson T., Scholten E.T., Schalekamp S., Schaefer-Prokop C.M., Philipsen R.H., Meijers A. (2020). COVID-19 on the chest radiograph: A Multi-Reader evaluation of an AI system. Radiology.

[16] Wang X., Deng X., Fu Q., Zhou Q., Feng J., Ma H., Liu W., Zheng C. (2020). A Weakly-Supervised framework for COVID-19 classification and lesion localization from chest CT. IEEE Trans. Med Imaging.

[17] Zhang H., Zhang J., Zhang H., Nan Y., Zhao Y., Fu E., Xie Y.H., Liu W., Li W.P., Zhang H.J. (2020). Automated detection and quantification of COVID-19 pneumonia: CT imaging analysis by a deep learning-based software. Eur. J. Nucl. Med. Mol. Imaging.

[18] WHO (2020). Clinical Management of Severe Acute Respiratory Infection When Novel Coronavirus (2019-nCoV) Infection Is Suspected.

[19] Wang D., Hu B., Hu C., Zhu F., Liu X., Zhang J., Wang B., Xiang H., Cheng Z., Xiong Y. (2020). Clinical Characteristics of 138 Hospitalized Patients with 2019 Novel Coronavirus-Infected Pneumonia in Wuhan, China. JAMA.

[20] National Health Commision of the People’s Republic of China (2020). Diagnosis and Treatment Protocol for COVID-19 in China.

[21] Shan F., Gao Y., Wang J., Shi W., Shi N., Han M., Xue Z., Shen D., Shi Y. (2020). Abnormal lung quantification in chest CT images of COVID-19 patients with deep learning and its application to severity prediction. Med. Phys..

[22] Karimi R., Tornling G., Forsslund H., Mikko M., Wheelock Å.M., Nyrén S., Sköld C.M. (2014). Lung density on high resolution computer tomography (HRCT) reflects degree of inflammation in smokers. Respir. Res..

[23] Kauczor H.U., Heitmann K., Heussel C.P., Marwede D., Uthmann T., Thelen M. (2000). Automatic detection and quantification of ground-glass opacities on high-resolution CT using multiple neural networks: Comparison with a density mask. AJR Am. J. Roentgenol..

[24] Ippolito D., Ragusi M., Gandola D., Maino C., Pecorelli A., Terrani S., Peroni M., Giandola T., Porta M., Talei Franzesi C. (2021). Computed tomography semi-automated lung volume quantification in SARS-CoV-2-related pneumonia. Eur. Radiol..

[25] Culver B.H., Graham B.L., Coates A.L., Wanger J., Berry C.E., Clarke P.K., Hallstrand T.S., Hankinson J.L., Kaminsky D.A., MacIntyre N.R. (2017). Recommendations for a standardized pulmonary function report. An official american thoracic society technical statement. Am. J. Respir. Crit. Care.

[26] Shi H., Han X., Jiang N., Cao Y., Alwalid O., Gu J., Fan Y., Zheng C. (2020). Radiological findings from 81 patients with COVID-19 pneumonia in Wuhan, China: A descriptive study. Lancet Infect. Dis..

[27] Pan F., Ye T., Sun P., Gui S., Liang B., Li L., Zheng D., Wang J., Hesketh R.L., Yang L. (2020). Time course of lung changes on chest CT during recovery from 2019 novel coronavirus (COVID-19) pneumonia. Radiology.

[28] Xie L., Liu Y., Xiao Y., Tian Q., Fan B., Zhao H., Chen W. (2005). Follow-up study on pulmonary function and lung radiographic changes in rehabilitating severe acute respiratory syndrome patients after discharge. Chest.

[29] Gevenois P.A., De Vuyst P., De Maertelaer V., Zanen J., Jacobovitz D., Cosio M.G., Yernault J.C. (1996). Comparison of computed density and microscopic morphometry in pulmonary emphysema. Am. J. Respir. Crit. Care.

[30] Matsuoka S., Yamashiro T., Matsushita S., Kotoku A., Fujikawa A., Yagihashi K., Nakajima Y. (2015). Quantitative CT evaluation in patients with combined pulmonary fibrosis and emphysema. Acad. Radiol..

[31] Colombi D., Bodini F.C., Petrini M., Maffi G., Morelli N., Milanese G., Silva M., Sverzellati N., Michieletti E. (2020). Well-aerated lung on admitting chest CT to predict adverse outcome in COVID-19 pneumonia. Radiology.

[32] Nishiyama A., Kawata N., Yokota H., Sugiura T., Matsumura Y., Higashide T., Horikoshi T., Oda S., Tatsumi K., Uno T. (2020). A predictive factor for patients with acute respiratory distress syndrome: CT lung volumetry of the well-aerated region as an automated method. Eur. J. Radiol..

[33] Ackermann M., Verleden S.E., Kuehnel M., Haverich A., Welte T., Laenger F., Vanstapel A., Werlein C., Stark H., Tzankov A. (2020). Pulmonary vascular endothelialitis, thrombosis, and angiogenesis in COVID-19. N. Engl. J. Med..

[34] Kalef-Ezra J., Karantanas A., Tsekeris P. (1999). CT measurement of lung density. Acta Radiol..

[35] Robert H.B., Robert A.W., Kirk G., Drummond M.B., Mitzner W. (2015). Lung density changes with growth and inflation. Chest.

[36] Bao C., Liu X., Zhang H., Li Y., Liu J. (2020). Coronavirus disease 2019 (COVID-19) CT findings: A systematic review and meta-analysis. J. Am. Coll. Radiol..

[37] Zhu J., Huang W.-C., Huang B., Zhu Y., Jiang X.-J., Zou J.-N., Yang G., Wang Z., Ji T., Gu M.-M. (2020). Clinical characteristics and prognosis of COVID-19 patients with initial presentation of lung lesions confined to a single pulmonary lobe. Am. J. Transl. Res..

[38] Yu Q., Wang Y., Huang S., Liu S., Zhou Z., Zhang S., Zhao Z., Yu Y., Yang Y., Ju S. (2020). Multicenter cohort study demonstrates more consolidation in upper lungs on initial CT increases the risk of adverse clinical outcome in COVID-19 patients. Theranostics.

