# Supplementary material for: Well-Aerated Lung and Mean Lung Density Quantified by CT at Discharge to Predict Pulmonary Diffusion Function 5 Months after COVID-19
Source: Diagnostics (Basel). 2022 Nov 23;12(12):2921. doi: 10.3390/diagnostics12122921 (PMC9776504; doi:10.3390/diagnostics12122921)
Supplement: Supplementary file 1 [file diagnostics-12-02921-s001.zip › diagnostics-2003871-supplementary.pdf]

Figure S1. Residual plots of linear model for assumptions of normality, linearity and homogeneity of variance. Shapiro-Wilk's test for normality: P=0.561; White's test for heteroscedasticity: P=0.112

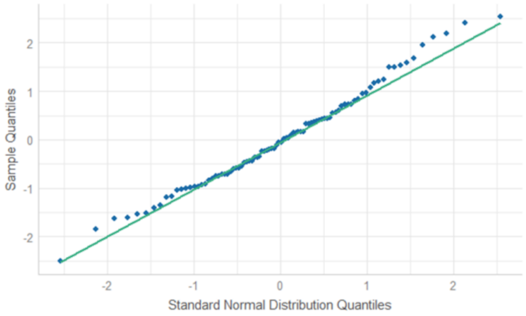

(a) Q-Q plot for normality of residuals

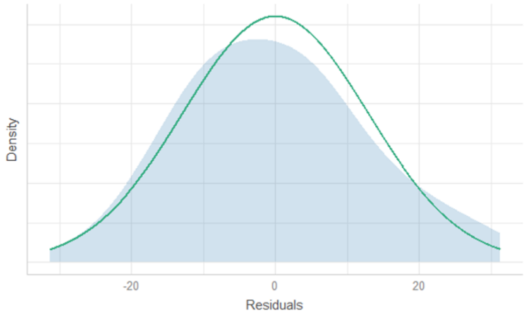

(b) Normal curve for residuals

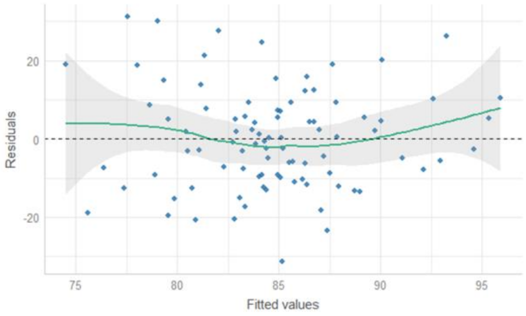

(c) Linearity of residuals

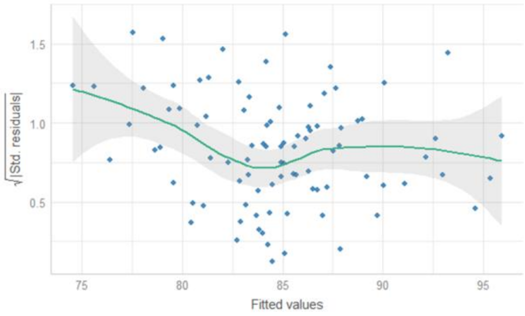

(d) Heteroskedasticity of residuals

Figure S2. Calibration plot of logistic model on mean lung density of total lung

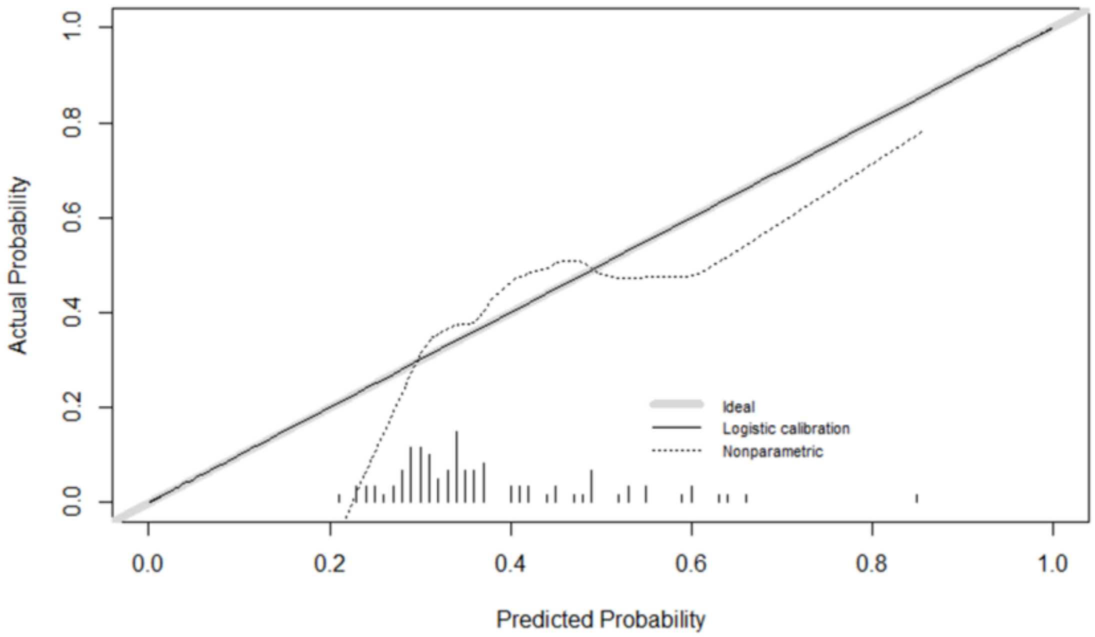

Nagelkerke's R^2	Hosmer-Lemeshow's test	Brier's score	AUCROC	Emax	E90	Eavg	Spiegelhalter's Z-test
0.073	5.847 (*p*=0.664)	0.223	0.644	0.251	0.124	0.052	0.058 (*p*=0.954)

AUCROC: area under the ROC curve; Emax: maximal absolute difference in predicted and loess-calibrated probabilities; E90: 0.9 quantile of absolute difference in predicted and loess-calibrated probabilities; Eavg: average of absolute difference in predicted and loess-calibrated probabilities.

Figure S3. Calibration plot of logistic model on mean lung density of left upper lobe

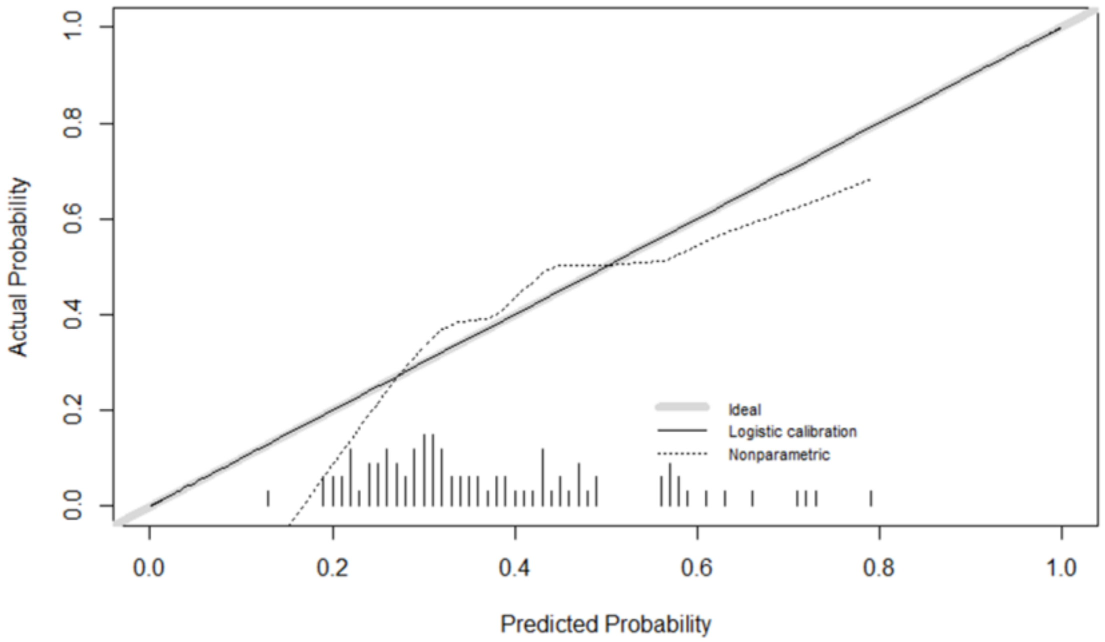

Nagelkerke's R^2	Hosmer-Lemeshow's test	Brier's score	AUCROC	Emax	E90	Eavg	Spiegelhalter's Z-test
0.113	5.458 (p=0.708)	0.216	0.680	0.216	0.088	0.048	0.066 (p=0.947)

AUCROC: area under the ROC curve; Emax: maximal absolute difference in predicted and loess-calibrated probabilities; E90: 0.9 quantile of absolute difference in predicted and loess-calibrated probabilities; Eavg: average of absolute difference in predicted and loess-calibrated probabilities.

Table S1. The lobar quantitative CT indices of COVID-19 patients at discharge

Characteristics	Left Upper Lobe (LUL)				Left Lower Lobe (LLL)			
	All patients （n=90）	Group1, abnormal diffusion function (n=34)	Group2, normal diffusion function (n=56)	*P* value	All patients （n=90）	Group1, abnormal diffusion function (n=34)	Group2, normal diffusion function (n=56)	*P* value
**MLD, HU**	-826.05 (-843.65, -792.43)	-806 (-833.7, -776.5)	-831.6 (-850.73, -800.63)	0.004*	-790.8 (-811.7, -755.15)	-786.9 (-810.63, -722.9)	-796.95 (-814.13, -761.35)	0.144
**LV, cm^3**	1012.65 (873.15, 1174.05)	974.7 (811.3, 1083.1)	1038.45 (888.13, 1224.83)	0.094	851.2 (683.48, 1120.6)	842.55 (683.48, 982.33)	855.15 (686.48, 1186.65)	0.659
**LV%**	25.08 (23.12, 27.36)	24.75 (22.32, 26.91)	25.27 (24.06, 27.5)	0.229	21.37 (18.88, 23.29)	22.21 (19.27, 24.28)	21.05 (18.53, 22.59)	0.223
**Well-aerated lung tissue**								
**WAL, cm^3**	845 (667.1, 941.4)	788.7 (638.85, 903.6)	854.4 (727.23, 1067.75)	0.043*	674.2 (476.15, 911.55)	677.9 (383.2, 735.58)	672.9 (494.6, 984.88)	0.356
**WAL%**	84.69 (76.98, 87.26)	79.18 (72.6, 85.71)	85.31 (79.93, 88.57)	0.004*	77.92 (66.47, 82.34)	76.47 (57.75, 81.99)	79.73 (71.75, 82.97)	0.133
**MLD_{Le}, HU**	-691.2 (-736.13, -549.93)	-692.3 (-734.75, -623.78)	-687.05 (-739.95, -495.85)	0.431	-649.15 (-733.8, -519.85)	-680.15 (-742.45, -562.35)	-642 (-728.55, -489.43)	0.211
**LeV, cm^3**	7.4 (0.2, 76)	35.2 (1.33, 114.73)	3.8 (0.1, 55.7)	0.011*	20.85 (3.15, 101.65)	44 (7.55, 119.4)	15.4 (1.1, 87.88)	0.101
**LeV%**	0.8 (0, 8.75)	3.15 (0.18, 13.48)	0.4 (0, 5.18)	0.009*	3.05 (0.4, 11.5)	4.3 (0.9, 12.58)	1.4 (0.15, 11.05)	0.124
**GGO**								
**GV, cm^3**	3.3 (0.1, 39.3)	13.85 (1, 61.98)	1.7 (0.1, 20.5)	0.010*	11.95 (1.95, 43.63)	16.75 (3.65, 56.03)	9.6 (0.6, 40.03)	0.119
**GV%**	45.92 (29.26, 58.86)	47.32 (39.64, 63.48)	44.41 (23.59, 56.23)	0.100	51.47 (32.76, 65.65)	47.74 (35.61, 65.95)	52.28 (29.89, 65.53)	0.726

Consolidation								
CV, cm^3	0.2 (0, 2.38)	0.6 (0.08, 5.58)	0.05 (0, 1.95)	0.020*	0.7 (0.08, 4.25)	1.1 (0.3, 3.38)	0.4 (0, 4.98)	0.207
CV%	1.43 (0, 4.61)	2.73 (0.59, 6.62)	0.27 (0, 3.94)	0.018*	2.61 (0.43, 7.37)	2.79 (1.06, 7.07)	1.84 (0, 8.1)	0.289

Characteristics	Right Upper Lobe (RUL)				Right Middle Lobe (RML)				Right Lower Lobe (RLL)			
	All patients（n=90）	Group1, abnormal diffusion function (n=34)	Group2, normal diffusion function (n=56)	*P* value	All patients（n=90）	Group1, abnormal diffusion function (n=34)	Group2, normal diffusion function (n=56)	*P* value	All patients（n=90）	Group1, abnormal diffusion function (n=34)	Group2, normal diffusion function (n=56)	*P* value
MLD, HU	-822.3 (-841.65, -799.4)	-818.3 (-834.5, -768.15)	-830.05 (-850.55, -805.2)	0.026*	-838.5 (-855.88, -806.88)	-829.6 (-847.33, -797.73)	-843.1 (-857.13, -815.75)	0.023*	-788.2 (-814.13, -736.98)	-782.35 (-806.18, -702.98)	-791.55 (-814.18, -761.4)	0.152
LV, cm^3	807.05 (691.05, 963.15)	765.25 (645.7, 884.63)	823.1 (722.35, 998.73)	0.074	421.9 (328.3, 493.25)	426.2 (334.53, 473.33)	416.1 (326.5, 507.63)	0.940	925.1 (761.28, 1098.75)	911.8 (740.78, 1035.75)	944 (762.13, 1127.78)	0.410
LV%	20.14 (18.38, 22.08)	20.47 (18.11, 22.11)	20.05 (18.64, 22.08)	0.717	10.18 (9.11, 11.45)	10.62 (9.4, 12.34)	10.07 (9.01, 11.07)	0.092	23.01 (19.93, 25.69)	23.3 (20.3, 25.85)	22.72 (19.93, 25.59)	0.829
Well-aerated lung tissue												
WAL, cm^3	684.7 (521.95, 824.8)	595.3 (492.95, 735.63)	710.5 (551.2, 870.33)	0.034*	355.85 (271.45, 435.83)	355.65 (263.83, 404.28)	359.6 (273.78, 455.35)	0.500	709.55 (538.25, 864.25)	692 (403.23, 852.6)	728.5 (567.83, 889.45)	0.291
WAL%	84.35 (78.59, 87.76)	82.55 (73.1, 86.57)	85.69 (79.99, 88.75)	0.012*	87.19 (80.9, 89.26)	85.83 (78.33, 88.41)	87.66 (81.9, 89.57)	0.016*	77.16 (63.67, 83.11)	76.2 (56.66, 82.43)	78.62 (70.19, 83.14)	0.252
MLD_{Le}, HU	-672.4 (-750.53, -537.1)	-688.3 (-753.88, -564.95)	-660 (-745.13, -450.9)	0.284	-681.7 (-749.38, 0)	-701.65 (-760.75, -568.05)	-669.15 (-744.13, 0)	0.182	-649.35 (-724.08, -519.93)	-676.4 (-727.28, -600.7)	-630.4 (-723.58, -474.88)	0.108
LeV, cm^3	13 (0.5, 91.68)	36.75 (2.75, 120.98)	6.55 (0.03, 65.2)	0.034*	1.05 (0, 13.03)	4.7 (0.18, 37.7)	0.45 (0, 4.1)	0.014*	43.25 (3.7, 149.4)	45.1 (8.88, 185.25)	43.25 (0.95, 141.25)	0.221
LeV%	1.5 (0.1,	4.45	0.6 (0,	0.025*	0.3 (0,	0.9 (0,	0.1 (0,	0.043*	4.85 (0.4,	5.7 (0.95,	4.65	0.284

	11.65)	(0.45, 15.43)	8.03)		4.05)	7.73)	1.95)		18.48)	29.43)	(0.13, 16)	
**GGO**												
**GV, cm^3**	5.35 (0.2, 36.35)	14.25 (0.98, 47.78)	3.45 (0.03, 27.03)	0.034*	0.4 (0, 4.73)	1.85 (0.1, 14.63)	0.2 (0, 2.03)	0.019*	24.75 (1.6, 59.3)	25.8 (5.78, 74.88)	23.75 (0.65, 57.6)	0.286
**GV%**	41.96 (24.5, 61.08)	50.16 (32.64, 62.92)	40.49 (4.67, 60.61)	0.166	32.93 (0, 50.05)	40.92 (17.26, 51.93)	30 (0, 49.41)	0.138	48.39 (30.92, 65.81)	55.02 (36.97, 66.42)	45.79 (26.76, 65.45)	0.169
**Consolidation**												
**CV, cm^3**	0.4 (0, 2.33)	0.9 (0.08, 3.8)	0.2 (0, 1.68)	0.050	0 (0, 0.4)	0.05 (0, 1.38)	0 (0, 0.1)	0.052	1.8 (0.1, 5.8)	1.55 (0.28, 9.93)	2.05 (0.03, 5.2)	0.498
**CV%**	1.42 (0, 3.87)	1.75 (0.54, 4.64)	1 (0, 3.21)	0.155	0 (0, 2.63)	0.24 (0, 2.67)	0 (0, 2.5)	0.113	3.3 (0.92, 9.35)	3.58 (1.36, 9.07)	2.85 (0.1, 9.35)	0.751

Note: Data are presented as medians (Q1, Q3). *P* values comparing with Group1 and Group2 are from Mann-Whitney *U* test.

* $P < 0.05$

Abbreviations: MLD: mean lung density; LV: each lobar volume; LV%: percentage of lobar volume to total lung volume; WAL: well-aerated lung tissue volume; WAL%: percentage of the well-aerated lung volume to each lobe; MLD_{Le}: mean lung density of lesion; LeV: total lung lesion volume; LeV%: percentage of lesion volume to each lobar volume; GV: ground-glass opacity volume; GV%: percentage of ground-glass opacity volume of the lesion; SCV: solid components volume; CV%: percentage of consolidation volume of the lesion.

Table S2. Linear regression analysis between lobar quantitative CT indices and DL_{CO}% of predicted

Variables	Univariable analysis										Multivariable analysis[a]	
	Left Upper Lobe (LUL)		Left Lower Lobe (LLL)		Right Upper Lobe (RUL)		Right Middle Lobe (RML)		Right Lower Lobe (RLL)			
	Coefficient (95% CI)	*P* value	Coefficient (95% CI)	*P* value	Coefficient (95% CI)	*P* value	Coefficient (95% CI)	*P* value	Coefficient (95% CI)	*P* value	Coefficient (95% CI)	*P* value
**MLD, HU**	-0.098 (-0.166, -0.03)	0.005*	-0.036 (-0.072, 0)	0.048*	-0.074 (-0.14, -0.008)	0.028*	-0.082 (-0.138, -0.026)	0.005*	-0.032 (-0.064, 0.001)	0.059	/	/
**LV, cm³**	0.011 (0.002, 0.021)	0.022*	0.009 (0, 0.018)	0.039*	0.015 (0.001, 0.028)	0.033*	0.014 (-0.009, 0.038)	0.230	0.011 (0.002, 0.02)	0.022*	/	/
**LV%**	0.006 (-0.576, 0.587)	0.984	0.061 (-0.527, 0.649)	0.838	-0.064 (-0.695, 0.568)	0.842	-0.937 (-2.141, 0.266)	0.125	0.211 (-0.454, 0.876)	0.530	/	/
**WAL, cm³**	0.014 (0.005, 0.023)	0.004*	0.011 (0.002, 0.019)	0.017*	0.018 (0.005, 0.03)	0.008*	0.024 (0, 0.047)	0.048*	0.011 (0.002, 0.019)	0.016*	/	/
**WAL%**	0.459 (0.152, 0.766)	0.004*	0.154 (-0.015, 0.322)	0.074	0.345 (0.075, 0.614)	0.013*	0.359 (0.102, 0.615)	0.007*	0.145 (-0.016, 0.306)	0.076	0.459 (0.152, 0.766)	0.004*
**MLD_{Le}, HU**	0.009 (-0.002, 0.02)	0.105	0.006 (-0.005, 0.018)	0.273	0.008 (-0.003, 0.018)	0.150	0.01 (0.002, 0.019)	0.021*	0.01 (-0.002, 0.021)	0.116	/	/
**LeV, cm³**	-0.041 (-0.073, -0.009)	0.012*	-0.033 (-0.065, -0.002)	0.040*	-0.031 (-0.059, -0.004)	0.027*	-0.098 (-0.172, -0.024)	0.010*	-0.023 (-0.046, 0)	0.052	/	/
**LeV%**	-0.402 (-0.694, -0.109)	0.008*	-0.201 (-0.398, -0.003)	0.046*	-0.253 (-0.456, -0.049)	0.016*	-0.359 (-0.637, -0.081)	0.012*	-0.18 (-0.331, -0.03)	0.020*	/	/
**GV, cm³**	-0.072 (-0.136,	0.030*	-0.056 (-0.116,	0.067	-0.052 (-0.101,	0.041*	-0.182 (-0.336,	0.022*	-0.04 (-0.084,	0.071	/	/

	-0.007)		0.004)		-0.002)		-0.027)		0.003)			
**GV%**	-0.038 (-0.149, 0.073)	0.500	-0.029 (-0.143, 0.084)	0.608	-0.085 (-0.196, 0.026)	0.131	-0.123 (-0.221, -0.025)	0.015*	-0.074 (-0.191, 0.044)	0.218	/	/
**CV, cm^3**	-0.302 (-0.6, -0.005)	0.047*	-0.121 (-0.274, 0.031)	0.117	-0.485 (-0.981, 0.012)	0.056	-0.368 (-0.768, 0.033)	0.071	-0.116 (-0.255, 0.024)	0.102	/	/
**CV%**	-0.382 (-0.739, -0.024)	0.037*	-0.287 (-0.58, 0.006)	0.055	-0.171 (-0.547, 0.204)	0.367	-0.627 (-1.136, -0.117)	0.017*	-0.178 (-0.508, 0.152)	0.287	/	/

[a]: In multivariable analysis, the predictor selected by linear regression was FLV% of left upper lobe.

* $P < 0.05$

Abbreviations: CI: confidence interval; MLD: mean lung density; LV: each lobar volume; LV%: percentage of lobar volume to total lung volume; WAL: well-aerated lung tissue volume; WAL%: percentage of the well-aerated lung volume to each lobe; MLD_{Le}: mean lung density of lesion; LeV: total lung lesion volume; LeV%: percentage of lesion volume to each lobar volume; GV: ground-glass opacity volume; GV%: percentage of ground-glass opacity volume of the lesion; CV: consolidation volume; CV%: percentage of consolidation volume of the lesion.

Table S3. Logistic regression analysis of predictors from lobar quantitative CT indices on diffusion dysfunction of convalescent COVID-19 patients

Variables	Univariable analysis															Multivariable analysis[a]		
	Left Upper Lobe (LUL)			Left Lower Lobe (LLL)			Right Upper Lobe (RUL)			Right Middle Lobe (RML)			Right Lower Lobe (RLL)					
	Coefficient (95% CI)	OR (95% CI)	*P* value	Coefficient (95% CI)	OR (95% CI)	*P* value	Coefficient (95% CI)	OR (95% CI)	*P* value	Coefficient (95% CI)	OR (95% CI)	*P* value	Coefficient (95% CI)	OR (95% CI)	*P* value	Coefficient (95% CI)	OR (95% CI)	*P* value
**MLD, HU**	0.016 (0.004, 0.027)	1.016 (1.004, 1.027)	0.008*	0.004 (-0.002, 0.01)	1.004 (0.998, 1.01)	0.192	0.012 (0.001, 0.022)	1.012 (1.001, 1.022)	0.031*	0.01 (0, 0.019)	1.01 (1, 1.02)	0.045*	0.004 (-0.002, 0.009)	1.004 (0.998, 1.009)	0.165	0.016 (0.004, 0.027)	1.016 (1.004, 1.027)	0.008*
**LV, cm^3**	-0.001 (-0.003, 0)	0.999 (0.997, 1)	0.065	-0.001 (-0.002, 0.001)	0.999 (0.998, 1.001)	0.401	-0.002 (-0.004, 0)	0.998 (0.996, 1)	0.062	-0.001 (-0.004, 0.003)	0.999 (0.996, 1.003)	0.719	-0.001 (-0.002, 0.001)	0.999 (0.998, 1.001)	0.307	/	/	/
**LV %**	-0.033 (-0.12, 0.054)	0.967 (0.887, 1.056)	0.459	0.024 (-0.063, 0.112)	1.025 (0.939, 1.118)	0.587	-0.032 (-0.134, 0.07)	0.969 (0.875, 1.072)	0.538	0.16 (-0.044, 0.364)	1.173 (0.957, 1.439)	0.125	0.018 (-0.083, 0.119)	1.018 (0.921, 1.126)	0.726	/	/	/
**WAL,**	-0.002 (-0.00	0.998 (0.99	0.022*	-0.001 (-0.002,	0.999 (0.99	0.	-0.003 (-0.005,	0.997 (0.995,	0.0	-0.002 (-0.005,	0.998 (0.995	0.3	-0.001 (-0.00	0.999	0.204	/	/	/

cm^3	4, 0)	6, 1)		0.001)	8, 1.001)	227	0)	1)	23*	0.002)	, 1.002)	27	2, 0)	(0.998, 1)				
WAL%	-0.066 (-0.118, -0.014)	0.936 (0.889, 0.986)	0.013*	-0.015 (-0.041, 0.01)	0.985 (0.96, 1.01)	0.240	-0.046 (-0.089, -0.002)	0.955 (0.915, 0.998)	0.039*	-0.037 (-0.079, 0.005)	0.964 (0.924, 1.005)	0.088	-0.016 (-0.04, 0.008)	0.984 (0.961, 1.008)	0.200	/	/	/
MLD$_{Le}$, HU	-0.002 (-0.004, 0)	0.998 (0.996, 1)	0.055	-0.001 (-0.003, 0.001)	0.999 (0.997, 1.001)	0.220	-0.001 (-0.003, 0)	0.999 (0.997, 1)	0.122	-0.001 (-0.003, 0)	0.999 (0.997, 1)	0.056	-0.002 (-0.004, 0)	0.998 (0.996, 1)	0.058	/	/	/
LeV, cm^3	0.005 (0, 0.01)	1.005 (1, 1.01)	0.064	0.004 (-0.001, 0.009)	1.004 (0.999, 1.009)	0.116	0.004 (0, 0.009)	1.004 (1, 1.009)	0.062	0.011 (-0.001, 0.022)	1.011 (0.999, 1.022)	0.076	0.002 (-0.001, 0.006)	1.002 (0.999, 1.006)	0.202	/	/	/
LeV %	0.049 (0.001, 0.098)	1.051 (1.001, 1.103)	0.047*	0.02 (-0.01, 0.05)	1.02 (0.99, 1.051)	0.198	0.036 (0.001, 0.071)	1.036 (1.001, 1.073)	0.045*	0.037 (-0.006, 0.08)	1.038 (0.994, 1.084)	0.093	0.019 (-0.004, 0.042)	1.019 (0.996, 1.043)	0.112	/	/	/
GV, cm^3	0.009 (-0.001, 0.019)	1.009 (0.999, 1.02)	0.084	0.005 (-0.004, 0.015)	1.005 (0.996, 1.015)	0.241	0.006 (-0.001, 0.014)	1.006 (0.999, 1.014)	0.111	0.017 (-0.006, 0.041)	1.017 (0.994, 1.042)	0.152	0.004 (-0.003, 0.01)	1.004 (0.997, 1.011)	0.244	/	/	/
GV %	0.016 (-0.001, 0.034)	1.017 (0.999, 1.035	0.069	0.006 (-0.011, 0.023)	1.006 (0.989, 1.024	0.48	0.014 (-0.004, 0.031)	1.014 (0.996, 1.032)	0.118	0.011 (-0.005, 0.026)	1.011 (0.995, 1.026)	0.167	0.018 (-0.002, 0.037)	1.018 (0.998,	0.073	/	/	/

		)			)	0								1.037)			
**SCV, cm^3**	0.092 (-0.024, 0.207)	1.096 (0.976, 1.231)	0.120	0.014 (-0.017, 0.044)	1.014 (0.983, 1.045)	0.376	0.058 (-0.021, 0.137)	1.06 (0.979, 1.147)	0.153	0.15 (-0.167, 0.468)	1.162 (0.846, 1.596)	0.354	0.015 (-0.014, 0.043)	1.015 (0.986, 1.044)	0.315	/	/	/
**SCV%**	0.059 (-0.009, 0.126)	1.061 (0.991, 1.135)	0.088	0.024 (-0.021, 0.069)	1.024 (0.979, 1.071)	0.299	0.034 (-0.022, 0.091)	1.035 (0.978, 1.095)	0.230	0.081 (-0.021, 0.182)	1.084 (0.98, 1.199)	0.118	0.003 (-0.045, 0.052)	1.004 (0.956, 1.054)	0.889	/	/	/

[a]: In multivariable analysis, the predictor selected by logistic regression was MLD of left upper lobe.

* $P < 0.05$

Abbreviations: OR: odds ratio; CI: confidence interval; MLD: mean lung density; LV: each lobar volume; LV%: percentage of lobar volume to total lung volume; WAL: well-aerated lung tissue volume; WAL%: percentage of the well-aerated lung volume to each lobe; MLD_{Le}: mean lung density of lesion; LeV: total lung lesion volume; LeV%: percentage of lesion volume to each lobar volume; GV: ground-glass opacity volume; GV%: percentage of ground-glass opacity volume of the lesion; CV: consolidation volume; CV%: percentage of consolidation volume of the lesion.
